# Combined delivery of small molecule and protein drugs as synergistic therapeutics for treating corneal neovascularization by a one-pot coassembly strategy

**DOI:** 10.1016/j.mtbio.2022.100456

**Published:** 2022-10-08

**Authors:** Wenjuan Huang, Liwen Wang, Ruhui Yang, Ronggui Hu, Qinxiang Zheng, Xingjie Zan

**Affiliations:** a Taizhou Hospital of Zhejiang Province Affiliated to Wenzhou Medical University, Linhai, Zhejiang Province, China; b School of Ophthalmology and Optometry, Eye Hospital, School of Biomedical Engineering, Wenzhou Medical University, Wenzhou, Zhejiang Province, China; c Huzhou Central Hospital, Affliated Central Hospital of Huzhou University, Huzhou, Zhejiang Province, China; d Department of Anesthesiology, Wenzhou Key Laboratory of Perioperative Medicine，the First Affiliated Hospital of Wenzhou Medical University, Wenzhou, Zhejiang, China; e State Key Laboratory of Molecular Biology, Shanghai Institute of Biochemistry and Cell Biology, Center for Excellence in Molecular Cell Science, Chinese Academy of Sciences, Shanghai, China; f The Affiliated Ningbo Eye Hospital of Wenzhou Medical University, Ningbo, China; g Wenzhou Institute, University of Chinese Academy of Sciences, Wenzhou China

**Keywords:** Hexahistidine-metal assembly (HmA), Protein drug, Codelivery, Combination therapy, Corneal neovascularization (CNV)

## Abstract

Combined drug administration is a potential strategy to increase efficacy through therapeutic synergy. Small molecule drugs and protein drugs are the two most popular kinds of drugs in medicine. However, efficiently encapsulating these two drugs still have key challenges due to their distinct properties (molecular weight, hydrophilicity, chemical groups, etc.), weak ability to penetrate through various biobarriers (cell membrane, endosome escape, tissue barriers dependent on the method of administration, etc.) and the easy deactivation of protein drugs during the construction of carrier and delivery process. Here, we utilize the hexahistidine-metal assembly (HmA), which can encapsulate a wide spectrum of drugs with high loading efficiency, to coencapsulate Dexp (a small molecule drug) and BVZ (protein drug) by a one-pot coassembly strategy. Our data demonstrated that Dexp and BVZ were coloaded into Dexp&BVZ@HmA with high efficiency, while the bioactivity of BVZ was well-maintained. Most importantly, when evaluating the therapeutic outcomes of drugs@HmA in a corneal neovascularization (CNV) model in vitro and in vivo, the combination group presented overwhelming efficacy compared to the monotherapy group. This strategy offers a platform to codeliver protein and small drugs and has the potential for treating anterior segment diseases as well as other diseases that need combination therapy.

## Graphical abstract

**Figure:**
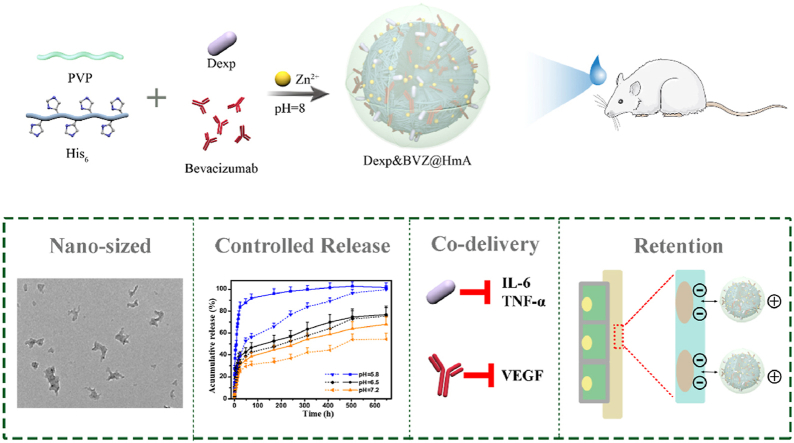

## 1 Introduction

Many diseases occur when one or more chained pathways of a complex biological regulatory process are malfunctioning or not working properly. In most cases, it is difficult to achieve the desired therapeutic effect by simply combating one specific pathway. Accumulative evidence has demonstrated that combined drug administration is a potential strategy to increase efficacy through therapeutic synergies [1,2]. Small molecule drugs and protein drugs are the two most popular kinds of drugs in medicine. The codelivery strategy of small molecule drugs and protein drugs showed promising therapeutic outcomes in cancer therapy and immunotherapy [3,4].

The cornea is the principle refractive component of the eye, and its transparency and avascularity are critical for the entry of light into the eye [5]. Corneal neovascularization (CNV), a sequelae of many corneal diseases that features new blood vessels erupting from the pericorneal vasculature, has become one of the major causes of blindness [6]. CNV patients suffer from decreased visual acuity caused by corneal scarring, edema, lipid deposition and inflammation, and these complications also affect neighboring tissues such as the iris [7]. Currently, CNV and its related diseases affect millions of people's daily lives [8], Vitar et al. reported a prevalence of 10.4% in a retrospective analysis [6]. Obviously, the prevention and treatment of CNV have become urgent issues that need to be addressed.

In healthy corneas, proangiogenic and antiangiogenic factors are well balanced to maintain avascularity. Unfortunately, under pathologic conditions, the balance is disturbed due to the predominance of proangiogenic factors, which initiates CNV through a very complicated biological process. Among these events, the recruitment of inflammatory cells is the beginning and core action [9]. Inflammatory cells secrete various proangiogenic factors and proteolytic enzymes, including major proangiogenic factors (of the vascular endothelial growth factor (VEGF) family) and strong mediators (many cytokines and enzymes, such as IL-6, IL-8 and matrix metalloproteinases), which promote limbal vascular endothelial cell proliferation and migration [9]. In addition, secreted VEGFs also in turn promote the chemotaxis of monocytes, the production of B cells and macrophage recruitment, thus resulting in a process called the “immune amplification cascade” [[9], [10], [11]].

Based on the above pathological process, anti-inflammatory and anti-VEGF drugs are the most widely clinically applied medicines to treat CNV. Considering that CNV is initiated by the recruitment of inflammatory cells, controlling inflammation by small molecule hormone anti-inflammatory drugs was first chosen as the preferred treatment [9]. However, their fast pharmacodynamics, obvious side effects, and limited medication time greatly restrict the application of such drugs [12,13]. Compared to small molecular drugs, protein drugs (anti-VEGF drugs) are famous for their high specificity and low side effects but suffer from a short half-life and weak ability to penetrate biobarriers in the cornea [14]. In addition, the monoadministration of anti-VEGF drugs cannot fully inhibit CNV development because angiogenesis occurs in the late stage of CNV and continues to develop in the presence of evoked inflammatory cells. The combination of strategies for controlling early-stage inflammation by small molecular drugs and the subsequent angiogenesis by protein drugs such as anti-VEGF was demonstrated to be much more effective in generating many encouraging outcomes [15]. Moreover, codelivery therapy may allow for a lower concentration of each drug, which reduces dose-dependent side effects.

Topical administration has been recognized as the most dominant administration route to treat corneal disease due to its convenience and noninvasiveness [16]. However, only 1 %–7% of drugs instilled by eye drops can be adsorbed by the ocular tissue due to ocular barriers, such as tear turnover, nasolacrimal drainage, conjunctiva and cornea barriers [16]. Compared to small molecular drugs, these barriers are more detrimental to protein drugs [17]. It has been reported that bevacizumab (BVZ), an anti-VEGF drug, was detected merely on the very superficial layer of the intact corneal epithelium even at high-frequency topical administration [18]. The subconjunctival injection of anti-VEGF drugs had to be performed to enhance bioavailability in the combined strategy, with the disadvantages of poor patient compliance, increased risks of infection and more complications.

Formulating various drug delivery vehicles, such as hydrogen, micro/nanoparticles, and vesicles, has received increasing attention due to their abilities to alter the pharmacokinetics and tissue distribution of the original drug [[19], [20], [21]]. Accumulating evidence indicates that vehicles with the ability to increase the retention time on the ocular surface and penetrate across ocular biobarriers could greatly improve the ocular availability of topically administered drugs. The formulation conditions have to be elaborately selected to preserve the bioactivity of the loaded cargo. It is especially critical when formulating protein drugs since the bioactivity of proteins is highly sensitive to their surrounding microenvironment, which might lead to the loss of bioactivity during formulation and delivery. In addition, the huge difference in physicochemical properties (molecular weight, hydrophilicity, chemical groups, etc.) between small molecular drugs and protein drugs is another immediate challenge to be faced when these two kinds of drugs are co-formulated, particularly when aiming for high entrapment efficiency [22] Taking advantage of the wide spectrum of drugs encapsulated in hydrogels, an injectable thermally responsive hydrogel was demonstrated to codeliver dexamethasone phosphate (Dexp, a class of steroid hormones) and BVZ (a kind of anti-VEGF drug) for treating CNV [23]. Although the hydrogel extended the retention time of the encapsulated drugs on the ocular surface, its limited ability to cross ocular biobarriers led to unsatisfactory results.

To address the above issues, we utilized the hexahistidine-metal assembly (HmA) formed through coordinative interaction between side imidazole group of hexahistidine and metal ions at mild conditions, which displayed encapsulating a wide spectrum of drugs with high loading efficiency [24], to coencapsulate Dexp and BVZ by a one-pot coassembly strategy. In previous studies [25,26], HmA exhibited a promising candidate nanocarrier of Dexp and BVZ for treating disease in anterior segment. Here, our data demonstrated that Dexp and BVZ were coloaded into Dexp&BVZ@HmA with high efficiency, while the bioactivity of BVZ was well-maintained. HmA greatly improved the bioavailability of BVZ and Dexp. Most importantly, the combination group presented overwhelming efficacy compared to the monotherapy groups when evaluating the therapeutic outcomes in vitro and in vivo, which presented excellent outcomes for this combinatorial treatment of anterior segment diseases as well as other diseases.

## 2 Materials and methods

### 2.1 Materials

Zinc nitrate hexahydrate (Zn (NO_3_)_2_·6H_2_O ​≥ ​99%), hexa-histidine (His_6_) with purity ≥99%, polyvinylpyrrolidone (PVP, Mw∼58 ​k), sodium hydroxide, hydrochloric acid, Bistris, dexamethasone sodium phosphate (Dexp) and bevacizumab (BVZ) were purchased from Aladdin (Shanghai, China). 4-(2-hydroxyethyl)-1-piperazineethanesulfonic acid (HEPES) was obtained from Macklin (Shanghai, China). The Bradford protein quantification kit was purchased from Vazyme (Nanjing, China). EAhy926 ​cell lines were obtained from Fenghui (Hunan, China). The Valukine TM ELISA kit (Human IL-6, Mouse TNF-α) was purchased from Novus Biologicals (Littleton, CO, USA). Trypsin-EDTA solution, 0.25% (without phenol red), fetal bovine serum (FBS), optimal cutting temperature compound (OCT) and DAPI were purchased from Thermo Scientific. Forty percent formal dehydrated ethanol was provided by Jinshan (Wenzhou, China). Anti-CD31 antibody (ab119339), anti-VEGF-A antibody (ab1316) and goat anti-mouse IgG H&L (Alexa Fluor® 594) (ab150116) were purchased from Abcam. The 3D corneal model and the culture medium for the 3D corneal model were purchased from BioOcullar TM RHC.

### 2.2 Animals

Male Sprague‒Dawley rats (weight 180–220 ​g, n ​= ​40) were provided by the Experimental Animal Center of Wenzhou Medical University. The animals were kept under laboratory conditions and fed a standard pellet diet. All animal study protocols complied with the guidelines set by the Association for Research in Vision and Ophthalmology (ARVO) Statement on the Use of Animals in Ophthalmic and Vision Research and with the Guidelines of the Animal Experimental Committee of Wenzhou Medical University (Zhejiang Province, China). Prior to treatment, all animals were confirmed to be free from ocular diseases.

### 2.3 Synthesis and characterization of nanoparticles

Ninety-four microliters of Zn (NO_3_)_2_ (0.1 ​M) were added dropwise to 2 ​mL of a mixed solution (7.2 ​mg His_6_, and 10 ​mg PVP, 1.6 ​mg Dexp and/or 6 ​mg BVZ) buffered with 50 ​mM HEPES at pH 8.0. The whole encapsulating procedure was conducted at 4 ​°C under sonication. When the nanoparticles were generated, they were collected by centrifugation (12,000 ​rpm, 10 ​min) and washed three times with deionized H_2_O. The particles were stored at 4 ​°C and sonicated for 1 ​min before characterization and cellular testing.

The size and ζ potential of the HmA and Drugs@HmA nanoparticles were measured using a Zetasizer Nano ZS instrument (Malvern, UK). The encapsulation efficiency (*EE* %) and loading capacity (*LC* wt%) of Dexp and BVZ in the nanoparticles were determined by high-performance liquid chromatography (HPLC) and a Bradford protein quantification kit, respectively. The mobile phase for the HPLC analysis was composed of acetonitrile and water (40/60, v/v), and the eluent was detected by a DAD detector at 240 ​nm. The *EE* % and *LC* wt% were calculated according to the following formulas:

$$EE\;\%=\frac{actual\;drug\;encapsulated}{theoretical\;drug\;encapsulated}*100\%$$

$$LC\;wt\%=\frac{weight\;of\;drug\;in\;nanoparticles}{weight\;of\;nanoparticles}*100\%$$

The morphology of the nanoparticles was detected by a TEM unit (FEI Talos F200S microscope, USA). Fourier transform infrared (FTIR) spectra were obtained on an FTIR spectrometer (Tensor II Bruker, Germany) using the KBr pellet method (sample: KBr ​= ​1:100) in the range of 4000–400 ​cm^−1^. Circular dichroism (CD) spectra were studied on a Chirascan-plus circular dichroism spectrometer (Applied Photophysics Ltd., UK). Measurements were conducted in a quartz cell with a 2 ​mm path length over the range of 190–300 ​nm under a nitrogen atmosphere.

The experiments above were assayed with three replicates and repeated three times.

### 2.4 In vitro drug release study

The in vitro drug release study of Dexp&BVZ@HmA was conducted in Bistris at different pH values. In brief, 1.7 ​mL of Dexp&BVZ@HmA (Dexp: 0.8 ​mg/mL; BVZ: 3 ​mg/mL) was added to a dialysis bag (MWCO 3.2 ​kDa or 1000 ​kDa), followed by the addition of 1 ​mL Bistris (10 ​mM, pH ​= ​5.8, 6.5 or 7.2) as a release medium for the duration of the in vitro release study. The above mentioned dialysis bags were placed in a centrifuge tube containing 37.3 ​mL of Bistris solution at the corresponding pH. At a predetermined time point, 1 ​mL of the test solution was withdrawn and replaced with the same volume of fresh medium. HPLC and Bradford protein quantification kits were applied to quantify the amount of Dexp and BVZ, respectively, that had been released. The cumulative release (%) at each time point was calculated according to the following formula:

$$Cummulative\;released\;drug\;amount\%=\frac{C_{t}*V_{t}+\Sigma_{i}\;C_{i}*V_{i}}{W_{total}}*100\%$$

where Ct and Ci are the concentration of the drug in the mother medium at testing time point (t) and the concentration of the drug in the sample drawn at testing time point (i) prior to t, respectively, Vt is the volume of the mother medium at time t, Vi (1 ​mL at each tested point) is the volume of medium withdrawn at time point (i), and Wtotal is the total weight of the drug encapsulated in the HmA particles. The above experiments were repeated three times.

### 2.5 Test of the bioactivity of BVZ

The 100 ​μL BVZ@HmA (BVZ: 3 ​mg/mL) and Dexp&BVZ@HmA (Dexp: 0.8 ​mg/mL, BVZ: 3 ​mg/mL) nanoparticles was treated by 200 ​μL 10 ​mM EDTA at pH ​= ​7.4 to disassemble the particles. Once the solution became clear, the concentration of released BVZ was quantified according to reference curve of free BVZ, which was obtained by UV–vis as reported [27]. The bioactivity of the released BVZ was detected by a BVZ ELISA Kit. The protocol provided by the manufacturer was followed. Briefly, a solution of free BVZ or BVZ released from BVZ@HmA and Dexp&BVZ@HmA was added to an incubator. After 5 ​min, the solution was removed and washed three times with cold PBS, followed by the addition of a horseradish peroxidase (HRP)-labeled antibody against BVZ and 3,3′,5,5′-tetramethylbenzidine (TMB). After adding the stop solution, the color and the absorbance intensity were recorded by a photo and microplate reader.

### 2.6 In vitro anti-inflammation test

Briefly, EAhy926 ​cells were seeded at a density of 1 ​× ​10^5^ ​cells per well in 48-well plates and incubated for 24 ​h, followed by stimulation with 250 ​ng/mL lipopolysaccharide (LPS) for 3 ​h. The culture medium was then removed and replaced with fresh DMEM containing 200 ​ng/mL LPS and either Dexp@HmA (Dexp: 0.8 ​mg/mL), BVZ@HmA (BVZ: 3 ​mg/mL) or Dexp&BVZ@HmA (Dexp: 0.8 ​mg/mL, BVZ: 3 ​mg/mL) for 24 ​h. The supernatant was then collected from each well, and the levels of interleukin-6 (IL-6) and TNF-α were measured using ELISA kits (Novus Biologicals, USA). Each experiment was repeated 3 times.

### 2.7 Migration assay

An in vitro migration assay was carried out as reported earlier [28]. Briefly, 2 ​× ​10^5^ EAhy926 ​cells were seeded in each well of 12-well plates and incubated overnight for cell coverage getting more than 95%. The next day, a straight-line scratch (per well) was made on a monolayer of EAhy926 ​cells with a sterile 100 ​μL pipet tip, and the debris was cleared by gentle washing with 1 ​× ​PBS. Different formulations (incubation medium alone or with Dexp@HmA (Dexp: 0.8 ​mg/mL), BVZ@HmA (BVZ: 3 ​mg/mL) or Dexp&BVZ@HmA (Dexp: 0.8 ​mg/mL, BVZ: 3 ​mg/mL)) were added to 1 ​mL of media. The plate was then incubated in a tissue culture incubator at 37 ​°C for 24 ​h. Images of the scratches were acquired at 10× magnification before and after incubation (0 and 24 ​h) and further analyzed using ImageJ. Each experiment was repeated at least three times. The rate of cell migration was determined according to the following equation:

$$m\;\left(\%\right)=\left(1-\frac{n}{r}\right)\times 100\%$$

where m is the migration of EAhy926 ​cells, n is the width of the scratch at 24 ​h, and r is the initial scratch width.

### 2.8 Tube formation assay

The in vitro tube formation assay was performed as reported earlier [28]. In brief, 150 ​μL of Corning Matrigel matrix (Fisher Scientific, USA) was placed in each well of a 48-well plate and incubated at 37 ​°C for 1 ​h to form a gel. Then, 100 ​μL of 5 ​× ​10^4^ EAhy926 ​cells was transferred to each well with or without other formulations (saline (NS), Dexp@HmA (Dexp: 0.8 ​mg/mL), BVZ@HmA (BVZ: 3 ​mg/mL), Dexp&BVZ@HmA (Dexp: 0.8 ​mg/mL, BVZ: 3 ​mg/mL)). The plate was incubated for 6 ​h at 37 ​°C with 5% CO_2_. At this time, clear endothelial tube formation was observed. Phase contrast images were acquired at the center of each well using an inverted microscope at 10× magnification. The degree of tube formation was determined by counting the number of the branch points by ImageJ as previous reported [29]. Each experiment was performed in triplicate.

### 2.9 Permeation test of recombinant 3D multilayer corneal epithelial cells in vitro

The 3D corneal model purchased from Guangzhou Biocell Biotechnology Co., Ltd was cultured in a transwell well plate. BioGrowth cell culture medium containing free FITC-labeled BVZ (F-BVZ), Dexp@F-HmA, BVZ@F-HmA and Dexp&BVZ@F-HmA was added to the inner chamber, and free culture medium was added to the outer chamber. After another 24 ​h of incubation, the plate was removed, and the cells were washed with PBS three times. Then, the cells were fixed with 4% paraformaldehyde, stained with DAPI and imaged under confocal microscopy (LSCM, A1, Nikon).

### 2.10 Alkali-induced CNV and medication

The CNV model was induced by alkali burns in rats as reported previously [23]. Briefly, the animals were generally anesthetized by an intraperitoneal injection of 10% chloral hydrate and topically anesthetized with 0.5% proparacaine hydrochloride (Alcaine; Alcon, Fort Worth, TX, USA). A filter paper disc (3 ​mm diameter) soaked in 1 ​M NaOH was placed on the central cornea of the right eye for 40 ​s. Immediately after the injury, the eyes were rinsed with 30 ​mL PBS. After instilling Levofloxacin for infection prevention, the rats were randomized into four groups: (1) 0.9% saline group (NS); (2) Dexp@HmA group (Dexp: 0.8 ​mg/mL); (3) BVZ@HmA group (BVZ: 3 ​mg/mL); and (4) Dexp&BVZ@HmA group (Dexp: 0.8 ​mg/mL, BVZ: 3 ​mg/mL). Each group was treated three times daily using the designated formulation after alkaline injury for 7 consecutive days.

### 2.11 In vivo biocompatibility

The ocular surface biocompatibility was studied in SD rats by setting three groups ((1) Dexp@HmA group (Dexp: 0.8 ​mg/mL); (2) BVZ@HmA group (BVZ: 3 ​mg/mL); and (3) Dexp&BVZ@HmA group (Dexp: 0.8 ​mg/mL, BVZ: 3 ​mg/mL)) with 5 rats in each group. 50 ​μL solution of each group was injected into the conjunctival sac of the left eye. While the right eye received normal saline (0.9%, 50 ​μL) as the control. The solution was applied thrice daily for 7 days. On the 8th day, corneal tissue sections were obtained, and corneal histological changes were observed by hematoxylin-eosin staining.

### 2.12 Alkali-induced inflammatory response and CNV

The inflammatory index and neovascularization area were applied to measure the severity of the corneal inflammation and CNV. Rats were anesthetized and examined under a slit lamp on Days 3, 7, and 14 after alkali burn. All observations were recorded by a single experienced ophthalmologist who was blinded to the allocation of the animals in each group. The inflammation index was obtained according to Table 1 as previously reported [23]. The final inflammatory index was obtained by summing the scores assigned for the different parameters and dividing the result by 9.

**Table 1:** Grading standard of inflammatory index.

Ocular presentation		Score
Conjunctival hyperemia	absent	0
	present but less than 1 ​mm	1
	present between 1 and 2 ​mm	2
	present and more than 2 ​mm ​= ​3	3
Central corneal edema	absent	0
	present with visible iris Details	1
	present without visible iris Details	2
	present without visible pupil	3
Peripheral corneal edema	absent	0
	present with visible iris Details	1
	present without visible iris Details	2
	present without visible iris	3

The CNV area was calculated by the following formula:

$$S=\frac{C}{12}\times \pi \times \left[r^{2}-{\left(r-l\right)}^{2}\right]$$

where S is the area, C is the number of clock hours since onset of CNV, l is the vessel length from the limbus, and r is the radius of the cornea [23]. An image analyzer (Image-Pro Plus 6.0; Media Cybernetics) was used to measure the radius.

Rats included a minimum of four animals per group per replicate experiment.

### 2.13 Histopathology

On the seventh day after alkali burn injury, the rats were euthanized by air embolism. Four additional rats were euthanized in the same way on Day 14. The corneas of the animals were fixed in 10% formalin for 3 days, conventionally dehydrated in ethanol, embedded in paraffin blocks, and sliced sagittally into 5 ​μm thick pieces. The sections were stained with hematoxylin and eosin and examined by light microscopy.

### 2.14 Immunofluorescence assay

An immunostaining test was performed to evaluate the expression of VEGF-A and CD31. As mentioned above, the rats were sacrificed at 3 days, 7 days and 14 days after corneal injury and Avastin treatments. The eyeballs were fixed, embedded in OCT compound and cut into 5 ​μm sections. The samples were pretreated with 1% Triton and 5% goat serum solutions. Anti-CD31 antibody (1:500) and anti-VEGF-A antibody (1:500) were used as the primary antibodies to target CD31 and VEGF-A, respectively. Goat anti-mouse IgG H&L (Alexa Fluor® 594) (1:500) was used as the secondary antibody. After immunostaining, the samples were mounted with a ProLong® Gold anti-faDexp kit and imaged by LSCM.

### 2.15 Preocular retention time evaluation

The preocular retention characteristics of the nanoparticles were evaluated using a noninvasive fluorescence imaging system (PerkinElmer IVIS Lumina XRMS Series III American).

An alkali burn model of the cornea was made in the left eyes before live imaging in rats as reported previously. Before imaging, 10% chloral hydrate (0.3 mL/100 ​g) was injected intraperitoneally for anesthesia, and the head region was imaged using an imaging system equipped with filter sets (excitation/emission, 495/520 ​nm). Imaging was performed at 0 ​h, 0.5 ​h, 1 ​h, 2 ​h, and 3 ​h after one drop (20 ​μL) of the various formulations was instilled into the left eye.

### 2.16 Statistical analysis

Statistical analysis was performed using SPSS 19.0 (IBM, Armonk, NY, USA). In one-way ANOVA, ∗p ​< ​0.05, ∗∗p ​< ​0.01, ∗∗∗p ​< ​0.001 and ∗∗∗∗p ​< ​0.0001 represent statistically significant results. The data are presented as the mean ​± ​standard deviation (SD). Graphs were generated using Origin 2018 (OriginLab, Northampton, Massachusetts, USA).

## 3 Results and discussion

### 3.1 Particle synthesis

**Fig. 1:**
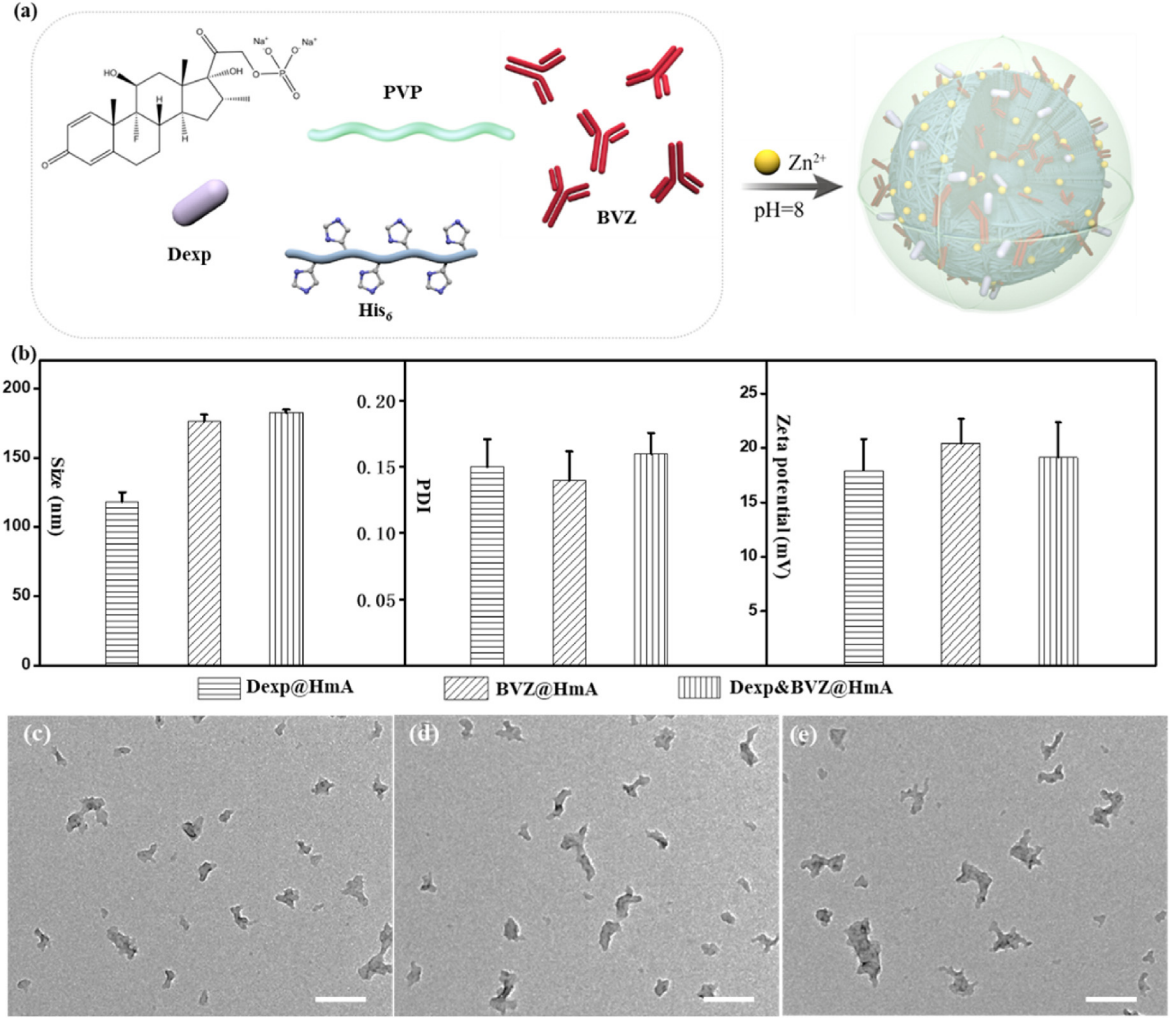
(a) Schematic presentation of the synthesis of Dexp&BVZ@HmA. (b) Size, PDI and zeta potential of Dexp@HmA, BVZ@HmA and Dexp&BVZ@HmA. TEM images of (c) Dexp@HmA, (d) BVZ@HmA and (e) Dexp&BVZ@HmA. Scale bar: 200 ​nm.

As shown in Fig. 1a, under ultrasonication at pH ​= ​8, Zn^2+^ was instilled into a mixture of PVP, Dexp, BVZ and His_6_ to generate Dexp&BVZ@HmA. Dexp@HmA and BVZ@HmA were produced by the same protocol but adding only one drug, Dexp or BVZ, to the mixture. Utilizing the weak coordinative interaction between PVP and Zn^2+^, the PVP acted as the surfactant to obtain well-dispersed nanoparticles as illustrated in other reports [30]. The size and zeta potential (ζ) of these particles were determined by dynamic light scattering (DLS). As shown in Fig. 1b, the mean sizes of Dexp@HmA, BVZ@HmA and Dexp&BVZ@HmA were ∼118 ​± ​10.3 ​nm, ∼176 ​± ​9.5 ​nm and ∼182 ​± ​6.6 ​nm, respectively, with polymer dispersity indices (PDI) of the different particles ranging from 0.14–0.16 and ζ potentials between +18.2 ​± ​2.8 ​mV and +19.1 ​± ​10.3 ​mV. The obviously increased sizes of BVZ@HmA and Dexp&BVZ@HmA might be attributed to the encapsulation of BVZ. The TEM images showed that the particles were relatively spherical with an irregularly shaped morphology (Fig. 1c–e). The smaller size in the electron micrograph images compared to the DLS results was attributed to dehydration.

### 3.2 Encapsulation and release behavior

**Fig. 2:**
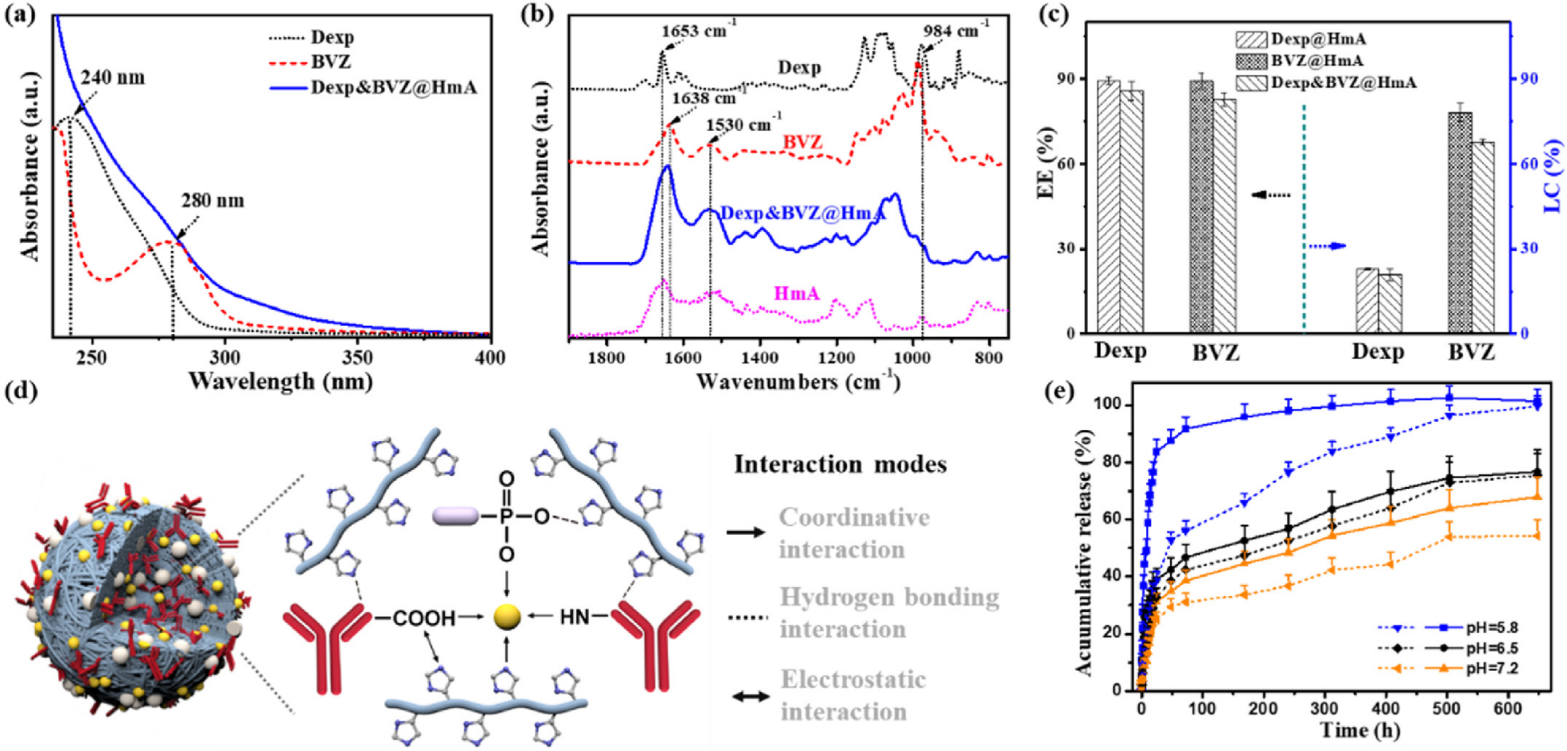
(a) UV–vis spectra and (b) FTIR spectra of Dexp, BVZ and Dexp&BVZ@HmA. (c) The encapsulation efficiency (*EE* %, left panel) and loading capacity (*LC* %, right panel) of Dexp and BVZ in Dexp&BVZ@HmA. (d) A schematic of the interaction forces in Dexp&BVZ@HmA. (e) Release profile of Dexp (solid line) and BVZ (dashed line) from Dexp&BVZ@HmA nanoparticles at pH 5.8, 6.5 and 7.2.

The encapsulation of Dexp and BVZ could be easily detected in the UV–vis spectra. As shown in Fig. 2a, the absorbances at 240 ​nm and 280 ​nm were attributed to Dexp and BVZ [25,26], respectively, which were observed in Dexp&BVZ@HmA. The encapsulation of Dexp and BVZ into HmA was also supported by the FTIR spectra. As shown in Fig. 2b (top curve), the Dexp characteristic peaks were present at 1653 ​cm^−1^ and 984 ​cm^−1^, which are assigned to the C＝O stretching vibration and the phosphate anion (P–O), respectively [31,32]. The amide-I region and amide-II region of BVZ shown in Fig. 2b (second curve from the top) exhibited their characteristic peaks centered at 1638 ​cm^−1^ and 1530 ​cm^−1^, respectively. In Fig. 2b (bottom curve), the characteristic peaks of HmA were very similar to that of BVZ, centering at 1638 ​cm^−1^ and 1530 ​cm^−1^. These characteristic peaks of both BVZ, Dexp and HmA were clearly displayed in Dexp&BVZ@HmA (Fig. 2b, second curve from the bottom), implying the successful encapsulation of BVZ and Dexp. The *EE* % of Dexp was determined by HPLC, and the BVZ was quantified by a Bradford protein quantification kit. As displayed in Fig. 2c (left panel), the *EE* % of Dexp was 89.4% for Dexp@HmA, and was 85% for Dexp&BVZ@HmA. The *EE* % of BVZ was 89.2% and 82% for BVZ@HmA and Dexp&BVZ@HmA, respectively. Another parameter that indicates the ability to load the drug, the loading capacity (*LC* %), was also investigated. In Fig. 2c (right panel), the *LC* % of Dexp in Dexp@HmA was 22.9%, and it was 20.9% in Dexp&BVZ@HmA. The *LC* % of BVZ was 78% in BVZ@HmA and 67.5% in Dexp&BVZ@HmA. Overall, the *EE* % and *LC* % were decreased in Dexp&BVZ@HmA compared to individually encapsulated nanoparticles (Dexp@HmA and BVZ@HmA). When comparing with the poly (d,l-lactide-*co*-glycolide)/polyethylenimine nanoparticles reported by Liu et al. which preloaded Dexp and postadsorbed BVZ by electrostatic interactions, Dexp&BVZ@HmA had almost the same encapsulation for BVZ and better *EE* % (85% vs. 56.9%) and *LC* % (20.9% vs. 9.3%) [33]. Such high *EE* % and *LC* % were due to the presence of multiple interaction modes inside the Dexp&BVZ@HmA nanoparticle. As schematically illustrated in Fig. 2d, in the framework constructed by the coordination between Zn^2+^ and His_6_, coordinative interactions between Zn^2+^ and the phosphate group of Dexp and between Zn^2+^ and the amino groups and/or carboxyl groups of BVZ played critical roles in wrapping Dexp and BVZ. The electrostatic interactions between the positively charged imidazole group of His_6_ and the negatively charged carboxyl groups of BVZ and between the imidazole group of His_6_ and the phosphate group of Dexp also participated. In addition, some weak interactions, such as hydrophobic-hydrophobic interactions between the imidazole group and the hydrophobic carbon backbone of Dexp and hydrogen bonding between the hydrogen bond donor of the imidazole group and the hydrogen bond acceptor of the phosphate group in Dexp, might take part in encapsulating Dexp and BVZ.

The in vitro release behavior was investigated at different pH values (pH ​= ​5.8, 6.5, 7.2) controlled by the buffer. As shown in Fig. 2e, the release of Dexp and BVZ from Dexp&BVZ@HmA was pH dependent. In detail, at pH 5.8, more than 80% of Dexp was released in the first 24 ​h, while at pH 7.2, only 25% was released; the release at pH 6.5 was between these two values. At the end of the experiment, all of the Dexp was released at pH 5.8, while only 53% was released at pH 7.2 and 70% was released at pH 6.5. The same pattern in the release profile at different pH values was found for the BVZ release. Notably, the burst release of BVZ was less than that of Dexp, and the release time seemed to last longer, which may be ascribed to the much larger molecular weight of BVZ (149 ​kDa) compared to Dexp (516 ​Da). Won and his colleagues synthesized a multishell rod to codeliver Dexp and BVZ to treat retinal vascular disease [34]. The release profile of BVZ presented a burst release with subsequent sustained release, while that of Dexp showed only a burst release. Since inflammation and neovascularization interact with each other, both Dexp and BVZ may require sustained release, which can be satisfied by Dexp&BVZ@HmA. This controlled release could be maintained in corneal inflammation since the tear fluid would be in an alkaline environment in this pathological condition [35], further presenting the potential of Dexp&BVZ@HmA to treat CNV.

### 3.3 Bioactivity of BVZ

**Fig. 3:**
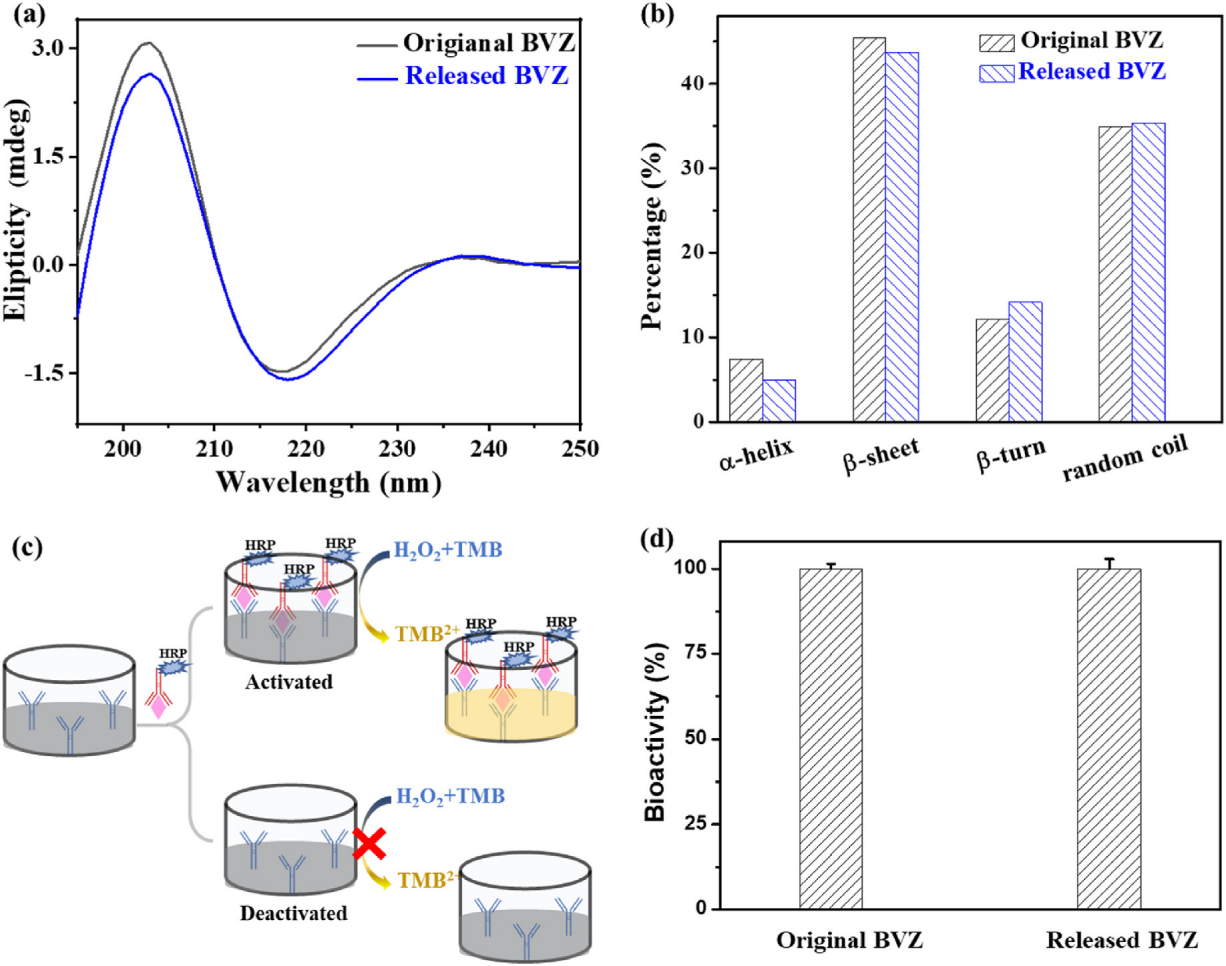
(a) Circular dichroism spectra of the original and released BVZs. (b) Circular dichroism spectroscopy analysis of the secondary structure of the original and released BVZs. (c) Schematic illustration of the working principle and operation process of the BVZ ELISA kit. (d) The bioactivity of the original and released BVZ tested by the BVZ ELISA kit.

The function of proteins is strongly dependent on their quaternary structure, which is closely related to their secondary structures. The integrity of the secondary structures of BVZ before and after release was assessed by circular dichroism (CD) spectroscopy. As shown in Fig. 3a, a negative band between 210 and 220 ​nm with a positive band at approximately 200 ​nm is a signature of a β sheet in BVZ. The sample released from Dexp&BVZ@HmA also showed this characteristic band, indicating that BVZ retained its secondary β-sheet structure after release from Dexp&BVZ@HmA particles. A detailed analysis of the compositions of the secondary structure is shown in Fig. 3b, in which no obvious change between the original BVZ and released BVZ was found, suggesting that the bioactivity of BVZ was preserved during the encapsulation and release processes. The BVZ ELISA kit was utilized to further determine the bioactivity of BVZ. Its working principle and operation process are shown in Fig. 3c. Briefly, the antibody against BVZ labeled with horseradish peroxidase (HRP) bound to BVZ was preattached onto the substrate, and then HRP catalyzed the oxidation process of 3,3′,5,5′-tetramethylbenzidine (TMB) under H_2_O_2_, with the color indicator changing from colorless to yellow. For deactivated BVZ, there is no binding between BVZ and the horseradish peroxidase (HRP)-labeled BVZ antibody, and therefore the subsequent discoloration reaction does not occur. The intensity of the color reflected the bioactivity of BVZ. As shown in Fig. 3d, the bioactivity of the released BVZ was the same as that of the original BVZ, which strongly indicated the preservation of BVZ bioactivity during the encapsulation and release processes.

### 3.4 *In vitro* anti-inflammatory efficacy

**Fig. 4:**
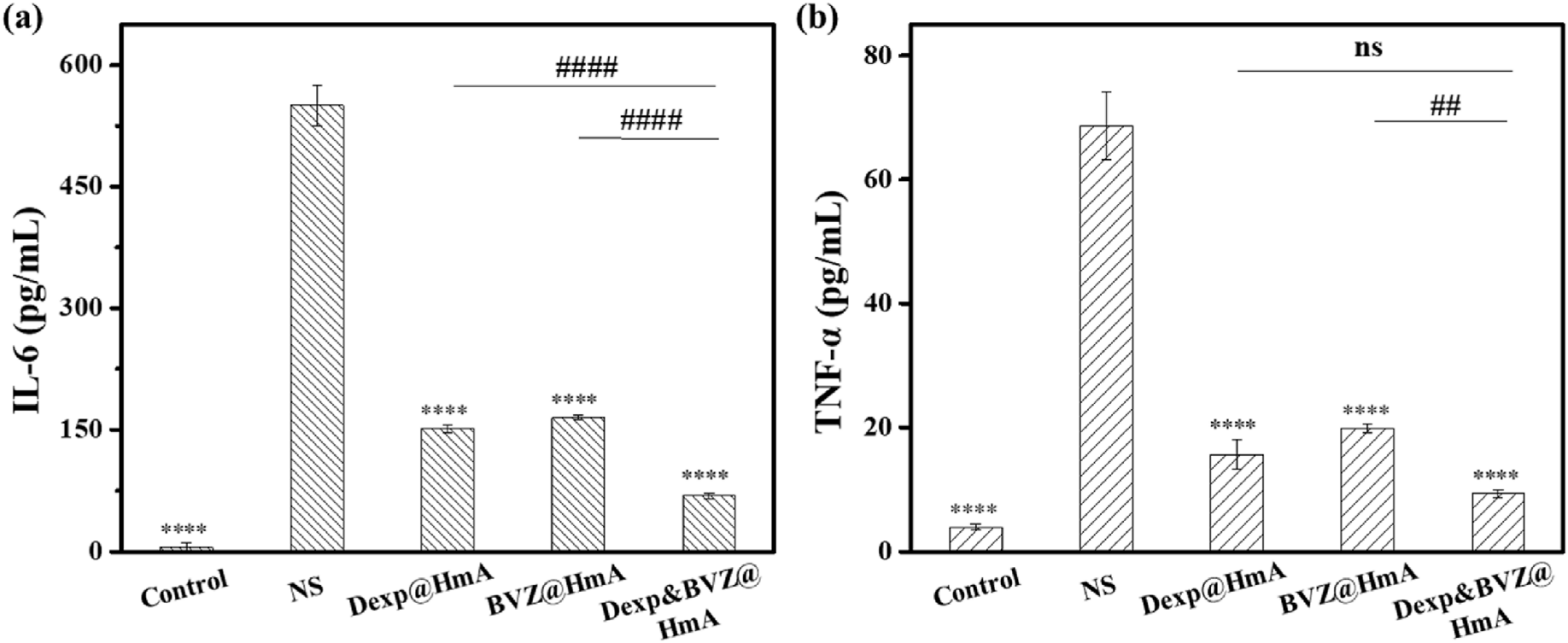
(a) IL-6 and (b) TNF-α in each group after treatment. ∗: difference between the NS group and the other group. ∗∗∗∗: p ​< ​0.0001; #: difference between the Dexp&BVZ@HmA group and the other group. ##: p ​< ​0.01, ####: p ​< ​0.0001.

Proinflammatory cytokines and chemokines are strong mediators of angiogenesis and are overexpressed in corneal inflammation [[36], [37], [38]]. Therefore, an inflammatory model was established in vitro to preliminarily evaluate the anti-inflammatory efficacy of Dexp&BVZ@HmA. To better mimic the process of neovascularization, EAhy926 endothelial cells were selected in this study since these cells were similar to vascular endothelial cells [39]. Cytokine production in EAhy926 ​cells was induced by LPS, and after treatment with different formulations, the levels of IL-6 and TNF-α were measured with an ELISA kit. As shown in Fig. 4a and b, the levels of cytokines (IL-6 or TNF-α) in the drug treatment groups (Dexp@HmA, BVZ@HmA and Dexp&BVZ@HmA) were much lower than those in the NS treatment group. The differences were statistically significant, indicating that both Dexp and BVZ work well after HmA encapsulation. Furthermore, the levels of IL-6 and TNF-α in the Dexp&BVZ@HmA group were the lowest, which suggests the superiority of combination therapy over monotherapy. The specific mechanism by which BVZ inhibits inflammatory factors is not clear, but the effect of BVZ in inhibiting inflammatory factors we found here is consistent with a previous report [40]. It had reported that EAhy926 ​cells expressed VEGF [41], which was inhibited by BVZ delivered by HmA, resulting in the reduced inflammatory level. Although there was no difference in the TNF-α levels between Dexp@HmA and Dexp&BVZ@HmA, this may be because the interaction between the inflammatory response and proangiogenic factors is not obvious in vitro experiments, and the advantages of the combination medication are not fully reflected.

### 3.5 *In vitro* antiangiogenic efficacy

**Fig. 5:**
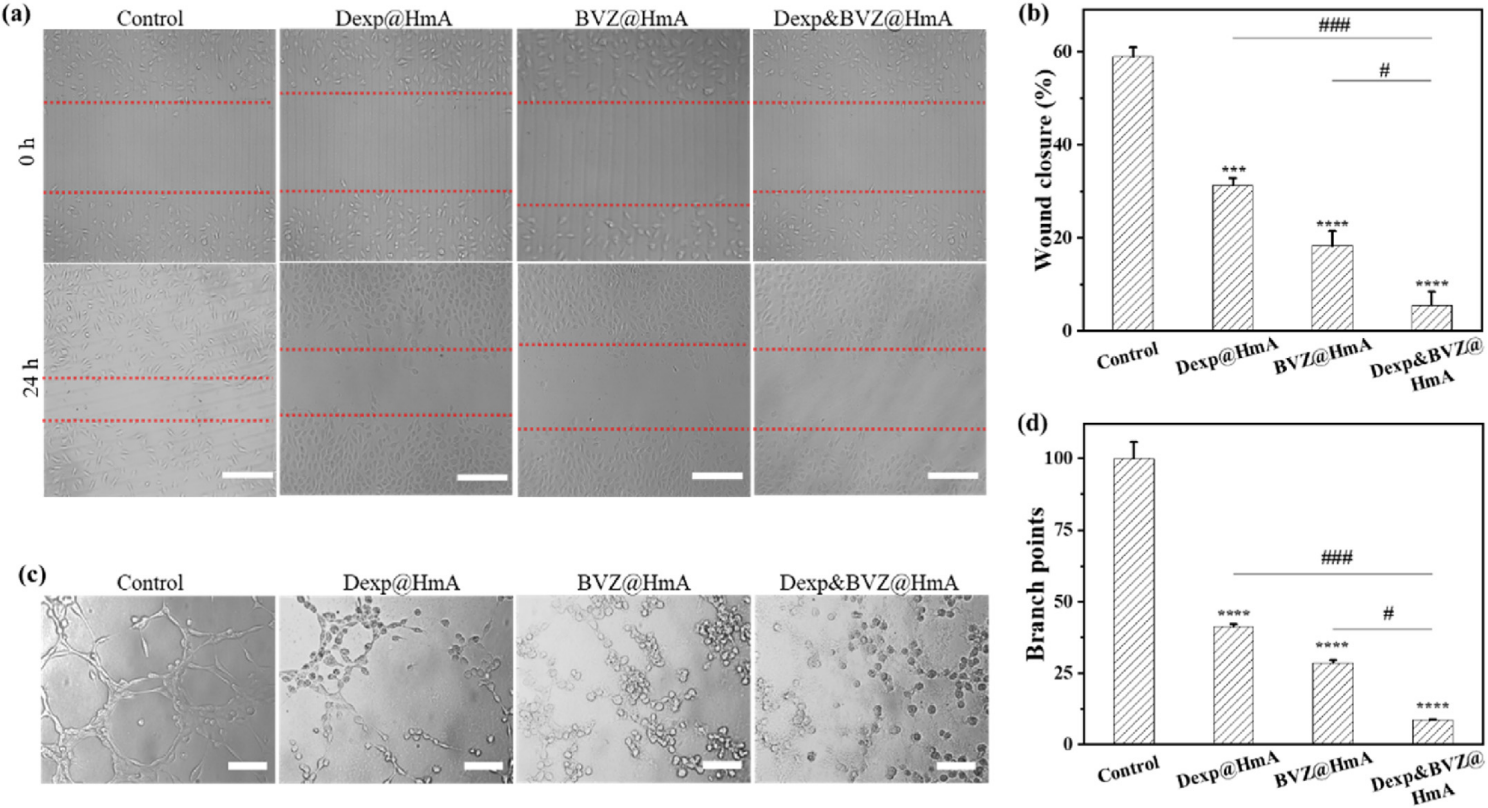
(a) Representative photographs from a wound healing assay were taken at 0 and 24 ​h after the generation of wounds in the monolayer cultures of EAhy926 ​cells using a pipet tip. Scale bar: 50 ​μm. (b) Representative images of tube-like structures of EAhy926 ​cells were acquired at 6 ​h after treatment with different formulations. Scale bar: 100 ​μm. Statistical results of (c) wound-healing assays and (d) tube formation assays on EAhy926 ​cells after different treatments. ∗: difference between the NS group and the other group. ∗∗∗: p ​< ​0.001; ∗∗∗∗: p ​< ​0.0001; #: difference between the Dexp&BVZ@HmA group and the other group. #: p ​< ​0.05; ##: p ​< ​0.01, ###: p ​< ​0.001.

Endothelial cell proliferation, migration and assembly form a solid cord that subsequently acquires a lumen [42]. Wound healing and tube formation assays were thereby carried out to evaluate the efficacy of Dexp&BVZ@HmA in attenuating in vitro neovascularization. As shown in Fig. 5a, the control cells migrated toward each other, with a decreasing open wound area, while the motility of the drug-treated EAhy926 ​cells was obviously inhibited. In detail, the migration of cells treated with Dexp&BVZ@HmA was totally inhibited, and the scratch area of cells exposed to monotherapy treatments decreased much more obviously than that of cells exposed to the combination therapy treatment. The closure area was quantified using ImageJ. As shown in Fig. 5b, the percentage of wound closure was lowest in Dexp&BVZ@HmA, and it was significantly different from either the control groups or the monotherapy groups. Representative photographs of the tube formation assay are presented in Fig. 5c. There were many tube-like structure networks in the control and NS treatment groups, and the tubular structure in the monotherapy groups was obviously less than that in the control and NS treatment groups. For Dexp&BVZ@HmA, most cells remained dormant, indicating that the EAhy926 ​cells were inhibited by Dexp&BVZ@HmA. We quantified the percentage of tube formation, as shown in Fig. 5d. The percentage of tube formation in the drug treatment groups (Dexp@HmA, BVZ@HmA and Dexp&BVZ@HmA) was much lower than that in the control or NS treatment groups, and the difference was statistically significant. Compared with the percentage of tube formation in the monotherapy groups (Dexp@HmA and BVZ@HmA), Dexp&BVZ@HmA showed a much lower rate of tube formation. These results are consistent with those of the wound healing assay. Based on the results of the anti-inflammation assay and anti-angiogenetic assay, Dexp&BVZ@HmA was the most efficacious, and the difference between the monotherapy treatments may be ascribed to the difference in the encapsulated drugs. Dexp is a first-line option in inflammatory treatment, so the level of IL-6/TNF-α was much more inhibited by Dexp@HmA than with BVZ@HmA. BVZ is a recombinant human monoclonal antibody against VEGF, so BVZ@HmA presented more potent efficacy in the anti-angiogenetic assay than Dexp@HmA.

### 3.6 In vivo evaluation of inflammation and CNV

Before administrating any drug, the biocompatibility of these nanoparticles was evaluated by H&E staining. As displayed in Fig. 5a, the tissue sections stained with H&E showed normal tissue structure and morphology after injecting Dexp@HmA, BVZ@HmA and Dexp&BVZ@HmA for 7 days (thrice daily). These results demonstrated that these particles had no or cytotoxicity.

**Fig. 6:**
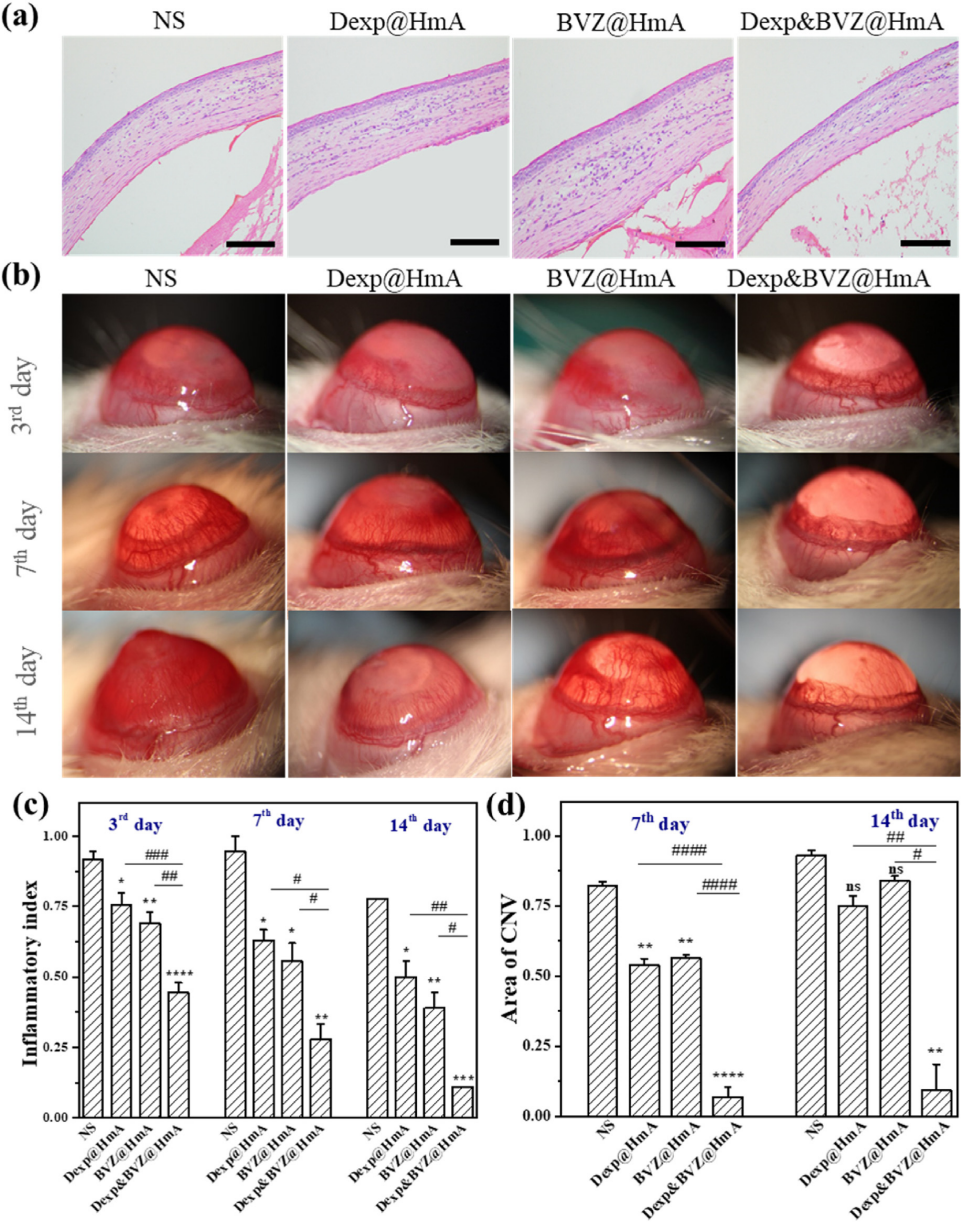
In vivo efficacy evaluation. (a) Histopathological staining of the ocular anterior segment after injecting 50 μL NS, Dexp@HmA, BVZ@HmA and Dexp&BVZ@HmA into the conjunctival sac of the left eye for 7 days (thrice daily). Representative photograph of the frontier segment: (b) lateral view. Statistical analysis of the (c) inflammatory index and (d) CNV area ratio. ns indicates no significant difference vs. control. ∗: difference between the NS group and the other group. ∗: p ​< ​0.05; ∗∗: p ​< ​0.01; ∗∗∗: p ​< ​0.001; ∗∗∗∗: p ​< ​0.0001; #: difference between the Dexp&BVZ@HmA group and the other group. #: p ​< ​0.05; ##: p ​< ​0.01, ###: p ​< ​0.001; ####: p ​< ​0.0001.

To investigate the efficacy of Dexp&BVZ@HmA in treating CNV, an alkali burned rat model was successfully created. Inflammation and neovascularization occurred successively after the injury. As shown in Fig. 6b, the lateral view of the frontier segment of the eye was observed by a slit lamp at postoperative days (PODs) 3, 7 and 14. The corneal tissue turned opaque, and edema was observed in all groups at POD 3. At PODs 7 and 14, the inflammation symptoms gradually alleviated, while neovascularization gradually became obvious. The inflammatory symptoms were quantified by the inflammatory index as described in the Methods section. As shown in Fig. 6c, at any time point, the inflammatory index was ranked as follows: NS ​> ​Dexp@HmA ​> ​BVZ@HmA ​> ​Dexp&BVZ@HmA. Except for NS on POD 3, the inflammatory index gradually decreased on POD 3, POD 7 and POD 14. The inflammatory index of the monotherapy groups (Dexp@HmA and BVZ@HmA) was also significantly decreased compared with that of the NS treatment groups. The inflammatory index of Dexp&BVZ@HmA was significantly lower than that of the other groups.

Small sprouts arising from preexisting limbal vessels could be observed at POD 3, and the length and density of the vessels gradually increased at POD 7 and POD 14. The severity of CNV was evaluated by comparing the neovascularization area between each group at PODs 7 and 14 (Fig. 6d). At POD 7, the drug-containing groups (Dexp@HmA, BVZ@HmA and Dexp&BVZ@HmA) significantly inhibited the growth of blood vessels compared with the NS-treated groups, implying that both Dexp and BVZ successfully inhibited CNV with HmA delivery. Furthermore, the CNV area in the Dexp&BVZ@HmA group was the lowest among all groups, and the difference was statistically significant (Dexp@HmA vs. Dexp&BVZ@HmA, p ​< ​0.0001; BVZ@HmA vs. Dexp&BVZ@HmA, p ​< ​0.0001). At POD 14, when compared with the CNV area in the NS-treated group, the drug-treated groups still showed a smaller area, but further statistical analysis showed no significance between the NS and monotherapy groups. However, the area in the combined medication groups was the smallest, with a significant difference between the other groups (NS vs. Dexp&BVZ@HmA, p ​< ​0.01 Dexp@HmA vs. Dexp&BVZ@HmA, p ​< ​0.01; BVZ@HmA vs. Dexp&BVZ@HmA, p ​< ​0.01). This result is somewhat different from that of the inflammation index, but for the two evaluation indicators, the best results were obtained in the Dexp&BVZ@HmA group, both indicating that the synergistic action of Dexp and BVZ with HmA delivery could lead to superior CNV inhibition.

### 3.7 Histopathology & immunohistochemistry

**Fig. 7:**
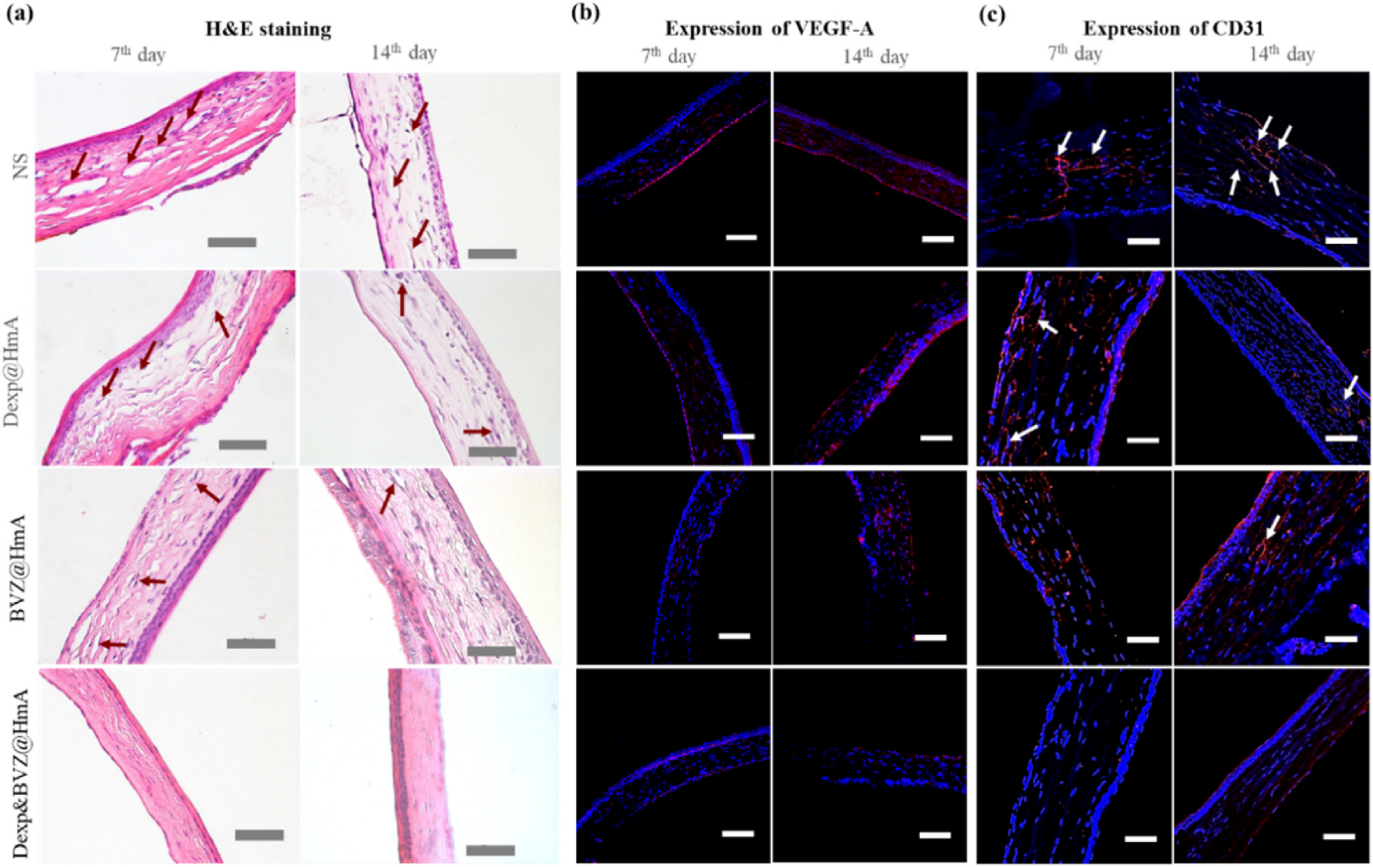
(a) Histological examination of corneal specimens by H&E staining. Arrows: vascular lumen. Scale bar: 50 ​μm. (b) Immunohistochemistry of VEGF-A expression in the corneal tissue after treatment with various formulations at different time points. VEGF-A (red); DAPI (blue); Scale bar: 100 ​μm. (c) Immunohistochemistry of CD 31 expression in corneal tissue after treatment with various formulations at different time points. CD 31 (red); DAPI (blue); Scale bar: 100 ​μm.

To further explore the efficacy of different formulations in attenuating inflammation and neovascularization, a histopathology examination and immunohistochemical staining were carried out. At POD 7, there was corneal stromal edema, and there were many apparent vascular lumens in the NS-treated group with a considerable amount of inflammatory cell infiltration (Fig. 7a). In the monotherapy groups (Dexp@HmA and BVZ@HmA), there were fewer apparent vascular lumens as well as fewer infiltrated inflammatory cells. For Dexp&BVZ@HmA, the stromal cells remained relatively compact with an almost invisible vascular lumen and inflammatory cells. At POD 14, the cornea turned relatively dense with less infiltration compared with that at POD 7 in each group. However, the vascular lumen in the NS and monotherapy groups showed no obvious decrease. In the Dexp&BVZ@HmA-treated group, the tissue section approached a normal cornea.

Vascular endothelial growth factor-A (VEGF-A) is considered a key regulator of blood vessel growth, with regard to either physiological angiogenesis or pathologic angiogenesis [11,17]. BVZ is a humanized monoclonal recombinant antibody that targets VEGF-A [43]. Therefore, we observed the expression of VEGF-A in the cornea by immunohistochemistry staining. As shown in Fig. 7b, at POD 3, obvious red fluorescence was found in the NS-treated group, suggesting potent expression of VEGF-A. Less red fluorescence was demonstrated in the monotherapy groups, implying that Dexp and BVZ partially inhibited the expression of VEGF-A. A very weak red fluorescent signal was observed in the Dexp&BVZ@HmA group, suggesting the efficient inhibition of VEGF expression by Dexp&BVZ@HmA. At POD 7, red fluorescence increased in each group but still ranked as follows: NS ​> ​Dexp@HmA ​> ​BVZ@HmA ​> ​Dexp&BVZ@HmA. At POD 14, the fluorescence signal in the NS- and monotherapy-treated groups increased, and the signal enhancement was stronger than that at POD 7. In contrast, Dexp&BVZ@HmA showed the weakest signal, which almost could not be observed. Moreover, the immunohistochemistry of CD 31, which is constitutively expressed on endothelial cells [44], was also conducted in our study. As shown in Fig. 7c, the weakest signal of CD 31 was found in Dexp&BVZ@HmA, indicating that Dexp&BVZ@HmA provided the best therapeutic effect either on POD 7 or POD 14. These results are consistent with those of histopathological examinations and clinical evaluations, further demonstrating the superiority of combination therapy.

### 3.8 Preocular retention

**Fig. 8:**
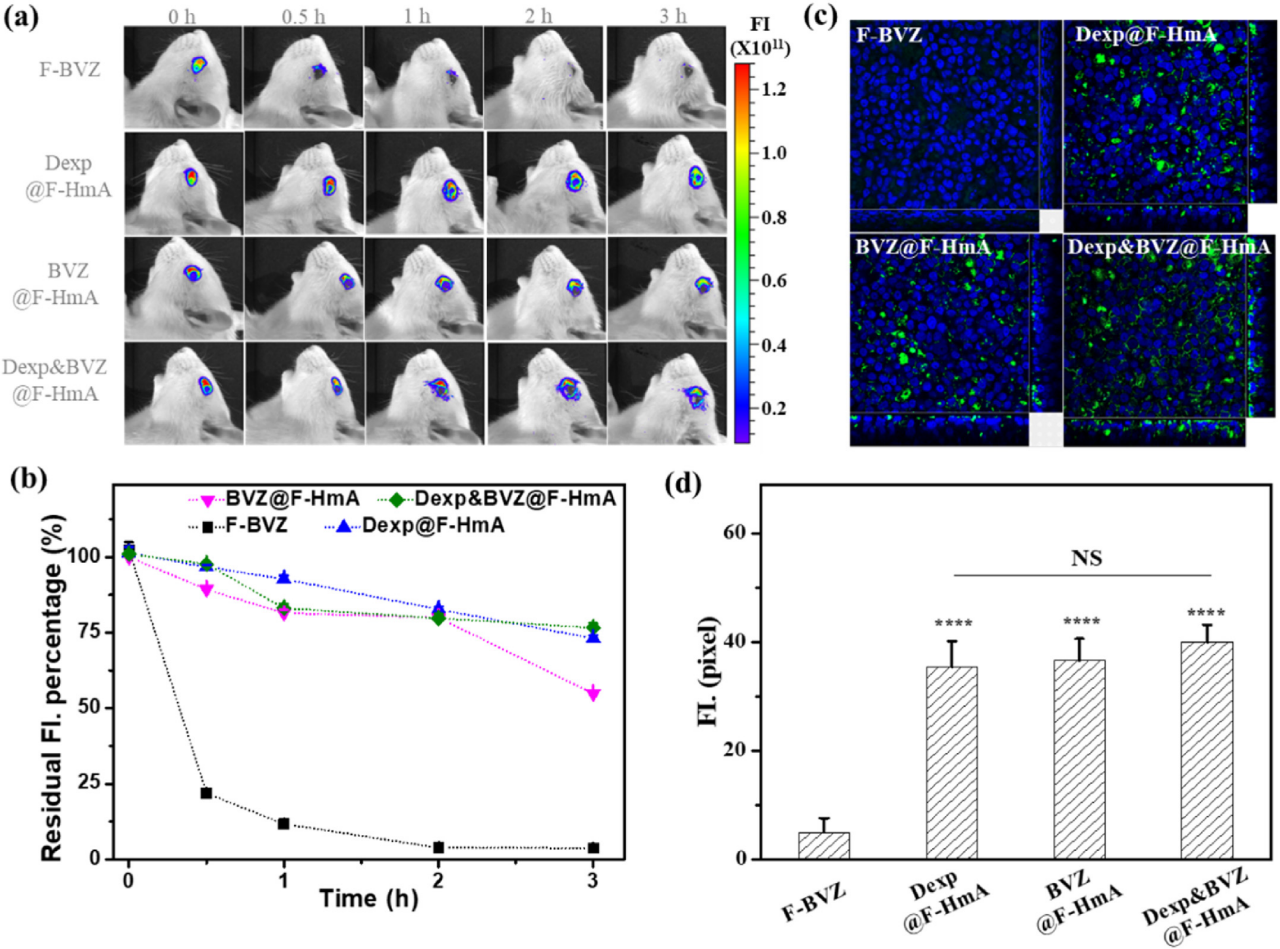
(a) Precorneal florescence images of various particles at different time points. (b) The residual fluorescence intensity (FI) percentage vs. time profiles for different particles. The residual fluorescence intensity was the total fluorescence intensity, which was obtained by affiliated software. (c) Typical 3D confocal fluorescent images of 3D multilayer corneal epithelium after coincubation with particles for 24 ​h. Orthogonal views from the x/z and y/z planes are displayed at the bottom and right side of each confocal image. (d) The statistical results of the averaged fluorescence intensity from at least 10 images in (c). The statistics were obtained using Image J.

To determine the reason for the improved bioavailability, the preocular retention time was evaluated by a multimmode optical live imaging system. The fluorescent imaging is shown in Fig. 8a. The total fluorescence intensity (FI) gradually decreased with time, indicating that the FITC-particles were gradually flushed away. Fig. 8b shows a more specific relationship between the residual fluorescence intensity percentage and time point. At 0.5 ​h after instillation, particles with F-HmA (HmA labeled with FITC) presented relatively strong fluorescence, while that in free F-BVZ (BVZ labeled with FITC) was sharply reduced. At the end of testing (3 ​h after instillation), drugs@F-HmA (Dexp@F-HmA, BVZ@F-HmA and Dexp&BVZ@F-HmA) retained more than 50% fluorescence, but it was close to zero for free F-BVZ. It is noteworthy that there were not significant differences between the drugs@F-HmA groups after 2 ​h of instillation. All these data suggested that the generated nanosized drugs@F-HmA contributed to the prolonged precorneal time. In addition, the ability of the vehicle to penetrate through biobarriers in the anterior segment was critical to the therapeutic efficacy of drug delivery. Among all of the biobarriers in corneal tissue, the corneal barrier caused by the tight junction between corneal epithelial cells is the major biobarrier. An in vitro 3D corneal model (BioOcullar TMRHC) was constructed and used to evaluate the precorneal penetrating ability of drugs@HmA, since it has a stratified structure and physiological and metabolic functions similar to those of the natural corneal epithelium. Compared with free F-BVZ, the drugs@HmA groups showed that green fluorescence was distributed throughout the 3D corneal model (Fig. 8c). The statistical analysis of the penetrated FI (Fig. 8d) suggested that there was no obvious difference between drugs@HmA, but drugs@HmA have a much stronger ability to pass through the 3D corneal model than free F-BVZ. Overall, drugs@HmA increased the precorneal retention time and the precorneal penetration of the drugs, which are keys to overcoming the anterior barrier in eye drop administration, as demonstrated in cumulative research [16]. The enhanced precorneal retention time and penetration ability might be ascribed to the nanosized and slightly positively charged characteristics of drugs@HmA. The slightly positive surface charge of drugs@HmA allows them to interact with negatively charged mucins on the ocular surface through electrostatic interactions, thus increasing their residence time on the ocular surface [45]. Additionally, the nanosize of drugs@HmA increased the corneal penetration ability, thereby increasing the bioavailability of eye drops.

After exploring the characterization and release behavior, the efficacy of drugs@HmA was evaluated in neovascular models in vitro and in vivo. In all evaluations, the combination group presented overwhelming efficacy compared to the monotherapy group. The combined medication allows treatment to be achieved at relatively lower concentrations. The better effect of combined administration may be due to the complex mechanism of neovascularization, as unilateral inhibition of a certain process is not enough to achieve the desired therapeutic effect. Additionally, the better therapeutic effect of the combination administration strategy offers a good opportunity to lower the administered dosage, which is highly valuable in reducing the side effects of small molecular drugs. This lower dosage is very important when administering eye drops, considering the convenience but low bioavailability of eye drops.

Last but not least is the issue of the biological toxicity of zinc ions. Zinc is an important trace metal element in humans and participates in various life activities. cell proliferation, growth, differentiation and apoptosis, gene expression and nucleic acid metabolism [46]. Zinc deficiency leads to a variety of metabolic diseases and organ dysfunctions [47]. Zinc ions are metabolized quickly and easily lost, and it is necessary to provide zinc ions in daily meals. Relative to the recommended daily intake of zinc ions in a healthy diet (12.5 ​mg/d for adult men and 7.5 ​mg/d for women), the administered amounts of zinc ions in our treatment (on the scale of several micrograms per day) were far below this standard. In addition, only a small amount of administered zinc ions are absorbed. In our previous reports, the cytotoxicity of HmA was very low, even when the concentration of HmA was as high as 100 ​mg/mL, and HmA did not cause toxicity to eye tissue during the seven-day instillation process [25]. Although these results all indicate that zinc ions in eye drops have very low toxicity for the human body, since the eye is a tissue rich in nerve cells, the toxicity of the long-term accumulation of metal ions is a problem that must be considered before any further clinical trial.

## 4 Conclusion

In summary, we synthesized nanosized and slightly positively charged drugs@HmA (Dexp@HmA, BVZ@HmA and Dexp&BVZ@HmA) with a narrow size distribution by one-pot coassembly strategy, into which Dexp and BVZ were effectively encapsulated. Drug@HmA displayed pH-dependent biphasic release of both Dexp and BVZ. Drug@HmA showed a prolonged precorneal retention time, enhanced precorneal penetration, and greatly improved bioavailability of Dexp and BVZ. Most importantly, the combination group presented overwhelming efficacy compared to the monotherapy groups in the in vitro and in vivo models. Such a strategy with elegant simplicity that produces surprisingly good therapeutic outcomes offers a platform to codeliver protein and small drugs and the potential for treating anterior segment diseases (such as glaucoma and endophthalmitis) as well as other diseases that need combination therapy.

## Credit author statement

Wenjuan Huang: Conceptualization, Methodology, Writing-Original draft preparation, Writing-Reviewing and Editing, Investigation. Liwen Wang: Data curation, Writing-Original draft preparation, Visualization. Ruhui Yang: Visualization, Investigation. Ronggui Hu: Software. Qinxiang Zheng: Conceptualization, Resources, Validation. Xingjie Zan: Conceptualization, Methodology, Writing- Reviewing and Editing.

## Declaration of competing interest

The authors declare that they have no known competing financial interests or personal relationships that could have appeared to influence the work reported in this paper.

## Data Availability

Data will be made available on request.
